# Undiagnosed hypertension and associated factors among adults in ethiopia: a systematic review and meta-analysis

**DOI:** 10.1186/s12872-023-03300-0

**Published:** 2023-05-27

**Authors:** Gizachew Ambaw Kassie, Afework Alemu, Amanuel Yosef Gebrekidan, Getachew Asmare Adella, Kirubel Eshetu, Abiyot Wolie Asres, Yordanos Sisay Asgedom

**Affiliations:** 1 School of Public Health, College of Health Science and Medicine, Wolaita Sodo University, Wolaita Sodo, Ethiopia; 2 School of Medicine, College of Health Science and Medicine, Wolaita Sodo University, Wolaita Sodo, Ethiopia; 3 School of Nursing, College of Health Science and Medicine, Wolaita Sodo University, Wolaita Sodo, Ethiopia

**Keywords:** Undiagnosed hypertension, Systematic review, Meta-analysis, Ethiopia

## Abstract

**Background:**

Hypertension is a major public health problem, resulting in 10 million deaths annually. Undiagnosed hypertension affects more people than ever before. It is more likely to be linked to severe hypertension, which can lead to stroke, cardiovascular disease, and ischemic heart disease. Thus, this systematic review and meta-analysis aimed to synthesize the prevalence of undiagnosed hypertension and its associated factors in Ethiopia.

**Methods:**

Databases, such as Medline/PubMed, Google Scholar, Science Direct, AJOL, and the Cochrane Library, were systematically searched to find potential studies published until December 2022. A Microsoft Excel spreadsheet was used to enter the extracted data. The pooled prevalence of undiagnosed hypertension and its associated factors was estimated using a random effect model. I^2^ statistics and the Cochrane Q-test were used to assess statistical heterogeneity across the studies. Begg’s and Egger’s tests were performed to identify possible publication bias.

**Results:**

A total of ten articles with 5,782 study participants were included in this meta-analysis. In the random effects model, the pooled prevalence of undiagnosed hypertension was 18.26% (95% CI = 14.94–21.58). Being older (OR = 3.8, 95% CI = 2.56, 5.66), having a body mass index > 25 kg/m2 (OR = 2.71, 95% CI = 2.1, 3.53), having a family history of hypertension (OR = 2.22, 95% CI = 1.47, 3.36), and having DM comorbidity (OR = 2.44, 95% CI = 1.38, 4.32) were significantly associated with undiagnosed hypertension.

**Conclusion:**

In this meta-analysis, the pooled prevalence of undiagnosed hypertension was found to be high in Ethiopia. Being older, having a BMI > 25 kg/m2, having a family history of hypertension, and having DM comorbidity were found to be risk factors for undiagnosed hypertension.

**Supplementary Information:**

The online version contains supplementary material available at 10.1186/s12872-023-03300-0.

## Introduction

Non-communicable diseases (NCDs) are widely acknowledged as a danger to socioeconomic progress. More than 50% of deaths worldwide in 2016 were due to cardiovascular issues, which were responsible for 72.3% of the NCD-related deaths. In low- and middle income nations where urbanization and lifestyle changes are developing quickly, NCDs are new priorities and exert extra pressure on health [1–3].

Hypertension is one of the most common NCDs worldwide, affecting millions of people. A lack of physical activity, smoking, excessive consumption of alcohol, low fruit and vegetable intake, and unhealthy eating habits are some of the major risk factors for hypertension. Approximately 1.28 billion people worldwide suffer from hypertension, with two-thirds living in low- or middle-income countries. A significant proportion of these individuals (46%) were unaware of their hypertension [4]. [5].

Sub-Saharan Africa (SSA) is one of the low and middle income Countries that have seen a shift in the burden of hypertension from high-income countries. With an estimated prevalence of 5–50%, SSA has been experiencing an exponential rise in hypertension over the last few decades. However, a sizable majority of those with hypertension are still undiagnosed, untreated, or only partially treated, which adds to the region’s growing CVD burden [4, 6, 7].

Undiagnosed hypertension is described as those who have hypertension but have not been informed of their condition by health professionals despite being hypertensive. It is a silent but deadly condition that can lead to serious health consequences, including heart disease, stroke, kidney disease, and cognitive impairment. Despite the availability of effective treatments, undiagnosed hypertension remains a major health concern owing to its lack of symptoms and low awareness of its potential health risks [8, 9]. One in every three adults has hypertension, and more than half are unaware of it[10, 11]. The prevalence of undiagnosed hypertension is 30% in sub-Saharan Africa. It is estimated that 73% of patients with hypertension are unaware of their condition. According to results from the World Health Organization STEP-wise approach to NCD risk factor surveillance (STEPS) study conducted in 2015, 15.8% of Ethiopians had hypertension and only a relatively small percentage of Ethiopians were aware that they had high blood pressure [12, 13]. Several studies in Ethiopia have also shown that undiagnosed hypertension is prevalent, with rates ranging from 8 to 35% [14–18].

A higher prevalence of undiagnosed hypertension in developing countries may be caused by a number of factors, including a lack of regular health screenings, restricted access to healthcare, low understanding of hypertension, and certain cultural practices and beliefs. In addition, people in these countries lack knowledge about hypertension, its management, and control strategies. Several studies indicated that undiagnosed hypertension is more common in people who are younger, have lower socioeconomic status, drink alcohol, are underweight, have no associated cardiovascular co-morbidities, and have no family history of the condition [19–21]. The reason behind this is that these groups may not seek medical attention as often, which would increases the prevalence of undetected hypertension [22].

The availability of systematically synthesized data on undiagnosed hypertension and its determinants, particularly in Ethiopia, is limited. The purpose of this review was to provide public health professionals with detailed knowledge that can be used to develop health promotion and disease prevention strategies and to enhance the early identification of adults at risk of hypertension in the future. Therefore, to highlight the demand for pertinent and useful control and prevention strategies, we conducted a systematic review and meta-analysis of the existing literature on the burden and determinants of undiagnosed hypertension in Ethiopia.

## Methods

### Searching strategy and study identification

A systematic review and meta-analysis were performed to estimate the magnitude of undiagnosed hypertension and its associated factors among adults in Ethiopia. We conducted a literature search on Medline, Google Scholar, Science Direct, AJOL, and the Cochrane library. All studies conducted on undiagnosed hypertension and underlying factors among adults in Ethiopia were retrieved. The search included all articles published until October 30, 2022. All searches were restricted to English-language studies. Medical subject heading (MeSH) (((((“Magnitude”) OR “prevalence” OR burden) AND “Undiagnosed hypertension”) OR “high blood pressure” OR undetected hypertension)) AND Ethiopia) were used in various combinations as the primary search keywords. During the systematic review and meta-analysis, we followed the Preferred Reporting Items for Systematic Reviews and Meta-Analyses (PRISMA) guidelines [23] (supplemental file). The research protocol was registered on PROSPERO under reference number CRD42022343672.

### Inclusion and exclusion criteria

All studies conducted in Ethiopia on the prevalence of undiagnosed hypertension among adults who were not diagnosed with hypertension and/or used anti-hypertensive medications were included. Furthermore, all original cross-sectional studies published in English and conducted across all regions of Ethiopia on adults were included in this systematic review and meta-analysis. Articles that had difficulty accessing the full text (after emailing the respective authors to obtain full texts) and studies that did not report the primary outcomes of interest were excluded.

### Outcome measurement

This systematic review and meta-analysis has two main objectives. The first objective was to estimate the pooled prevalence of undiagnosed hypertension among Ethiopian adults. The second objective was to identify factors associated with undiagnosed hypertension among adults in Ethiopia. Undiagnosed hypertension was defined as adults (age 18 and older) whose hypertension had not been diagnosed by a physician but whose blood pressure levels satisfied established criteria for hypertension, such as a blood pressure greater than or equal to > 140/80 as per the WHO classification. For second outcome, the odds ratio was used to measure the strength of the association between undiagnosed hypertension and the associated factors. The pooled odds ratio was calculated from the primary studies using two-by-two tables.

### Data abstraction

All retrieved studies from all databases were imported into Endnote version X7, and duplicate articles were manually removed. Two independent reviewers (GAK and AA) screened all articles for eligibility: first, the abstract and title, and then the full text. If conflicts over the choice of study could not be resolved, a third investigator was invited to mediate (GAA). Data extraction Excel spread sheet software was used to extract data from the included articles. The format for data extraction included the authors’ names, publication dates, research locations, regions, sample sizes, overall case counts, prevalence of undiagnosed hypertension, quality rating, and factors (age, sex, body mass index, family history of hypertension, sedentary life style, cigarette smoking and presence of DM comorbidity) associated with undiagnosed hypertension (a, b, c, and d).

### Quality assessments

To assess quality of each study Joanna Briggs Institute (JBI) tool adapted for cross-sectional studies was used to assess the quality of each study [24]. The following criteria for evaluation were included in the tool: appropriateness of the source population list, proper recruitment of study participants, sample size sufficiency, appropriateness of the study area and subject description, data analysis with sufficient coverage of the sampled data analysis, measurement of the condition using a standard, reliable, and consistent approach for all participants, suitability of statistical analysis, adequate response rate, or use of appropriate handling mechanism for low response rate. Two impartial reviewers critical appraised each study. Discussions among the reviewers were used to resolve disagreements. If the independent reviewers couldn’t reach an agreement, a third reviewer was consulted to resolve the conflict. Studies that received a final quality rating checklist score of 50% or higher were then included in this systematic review and meta-analysis.

### Statistical analysis

The data were entered into a computer using Microsoft Excel spreadsheet software and exported to STATA 14.1 software for analysis. The inverse variance (I^2^) test was used to assess the heterogeneity across studies with a p-value of 0.05. Inverse variance (I^2^) with Cochran’s Q statistic values of 0%, 25%, 50%, and 75% was assumed to represent no, low, medium, and high heterogeneity, respectively. A random effects meta-analysis model was used to estimate the pooled prevalence undiagnosed hypertension among adults (I^2^ > 91.3%, P = 0.01). Both Egger’s and Begg’s tests were used to look for evidence of publication bias, with a p-value of less than 0.05 used as a cut-off point. We also performed a leave-one-out sensitivity analysis to evaluate small study effects. By excluding each study one at a time, an analysis was performed to assess the effect of each study on the pooled prevalence of undiagnosed hypertension. The second outcome of this study was factors frequently associated with undiagnosed hypertension. For binary data, (determinants of undiagnosed hypertension), the input variables required by “metan” contained the cells of the 2 × 2 tables; i.e., the number of adults who had and who hadn’t undiagnosed hypertension in the exposed and non-exposed groups in each study. All potential determinants associated with undiagnosed were determined using the odds ratio (OR) and calculated based on the binary outcomes of the included primary studies. A random effect meta-analysis was used to estimate the pooled odds ratio with a 95% confidence interval. Finally, findings were presented in the form of forest plots with the corresponding effect size and 95% confidence intervals.

## Results

### Studies selection

**Fig. 1:**
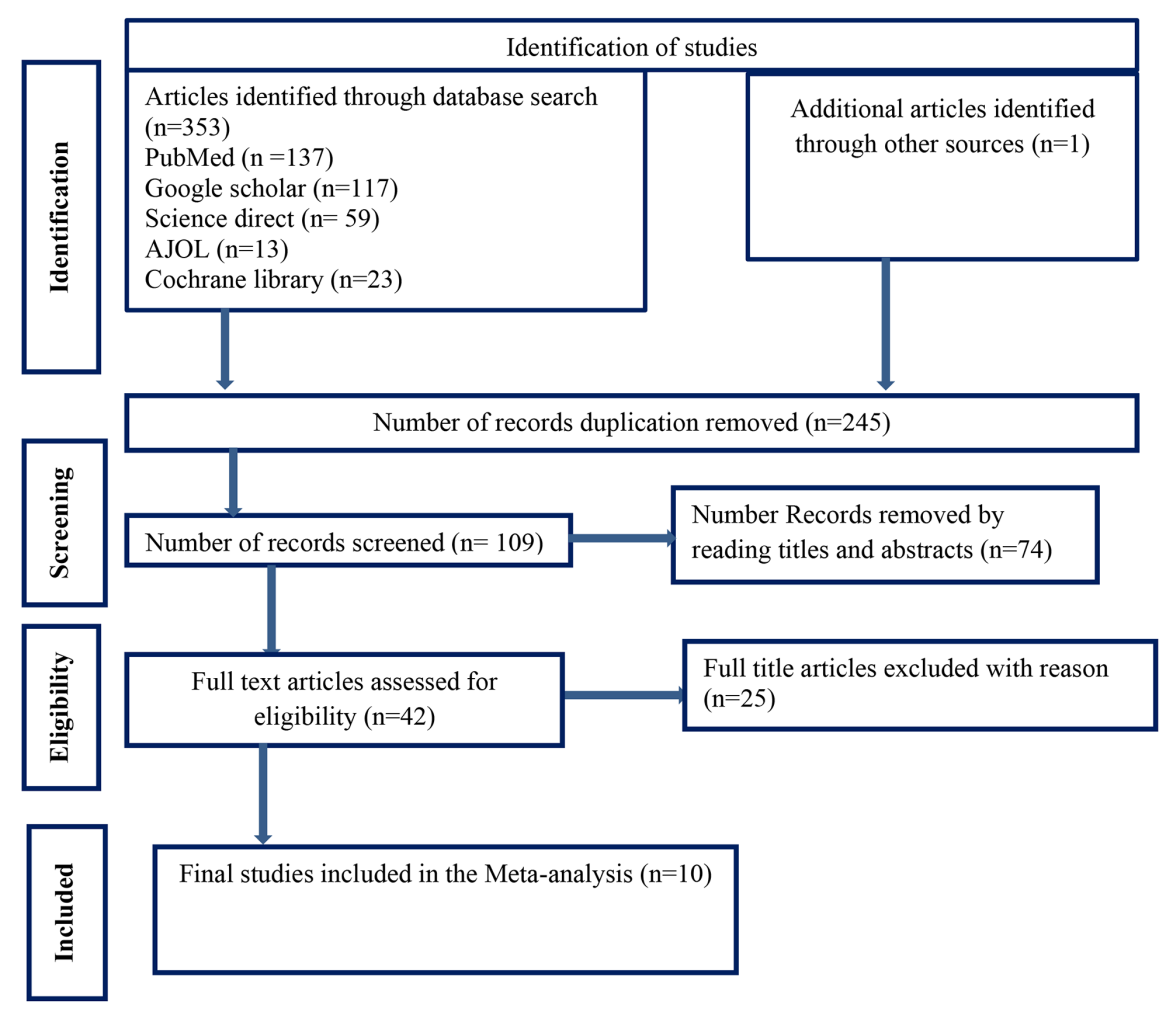
PRISMA flow diagram of articles screened and the selection process on undiagnosed hypertension and Associated Factors among adults in Ethiopia, 2023

Through database searches, 354 published and unpublished studies were identified. A total of 109 studies were included in the screening stage, while 245 duplicate studies were detected and eliminated. Forty two complete articles remained after 74 studies were removed based on the title and abstract screening. Ultimately, 10 studies that satisfied the eligibility criteria were considered for inclusion in the final analysis to estimate the pooled prevalence of undiagnosed hypertension and its determinants among adults in Ethiopia. The detailed selection procedure illustrated in (Fig. 1)

### Characteristics of included studies

A total of ten cross-sectional studies (5,782 adults) were included in this systematic review and meta-analysis. Two of the studies were institution-based cross-sectional studies [8, 25], while eight were community-based cross-sectional studies [26–33]. The publication period spanned from 2018 to 2022. The minimum and maximum sample sizes were 383 and 915 in the South Nations, Nationalities, and People’s Region (SNNPR) and Oromia, respectively [26, 27]. Regarding the distribution of studies across the country’s regions, two were from the Amhara region [8, 28], two from the Oromia region[25, 26], one from Sidama [27], one from Addis Ababa administrative city [29] and four from SNNP [30–33] (Table 1).

**Table 1:** Descriptive summary of 10 studies included in the meta-analysis of undiagnosed hypertension among adults in Ethiopia, 2023

No	Authors	Year	Mean age	Study area	Study setting	Region	Study design	Sample size	Prevalence (%)
1	Mogas et al. (25)	2021	35	Jimma	Urban	Oromia	CS	915	21.2
2	Dejene et al(8)	2021	34.1	Debre Markos	Urban	Amhara	CS	513	24.8
3	Getachew et al(29)	2018	33	Addis Ababa	Urban	AA	CS	422	13.3
4	Wachamo et al(26)	2020	33.5	Hawassa	Urban	Sidama	CS	383	12.3
5	Ayalew et al. (30)	2022	39.2	wolaita sodo	Urban	SNNPR	CS	644	28.8
6	Essa et al. (27)	2022	36.32	Debre Markos	Urban	Amhara	CS	600	12.7
7	Haligamo et al. (33)	2021	34.5	Gunchire	Rural	SNNPR	CS	574	15.3
8	Elias et al. (31)	2022	38.85	Mizan Aman	Urban	SNNPR	CS	738	14.8
9	Gelassa et al(32)	2022	37.2	Dano district	Rural	SNNPR	CS	605	21.32
10	Zawudie et al. (28)	2022	39	Butajira	Urban	SNNPR	CS	388	18.8

### Pooled prevalence of undiagnosed hypertension among adults in Ethiopia

**Fig. 2:**
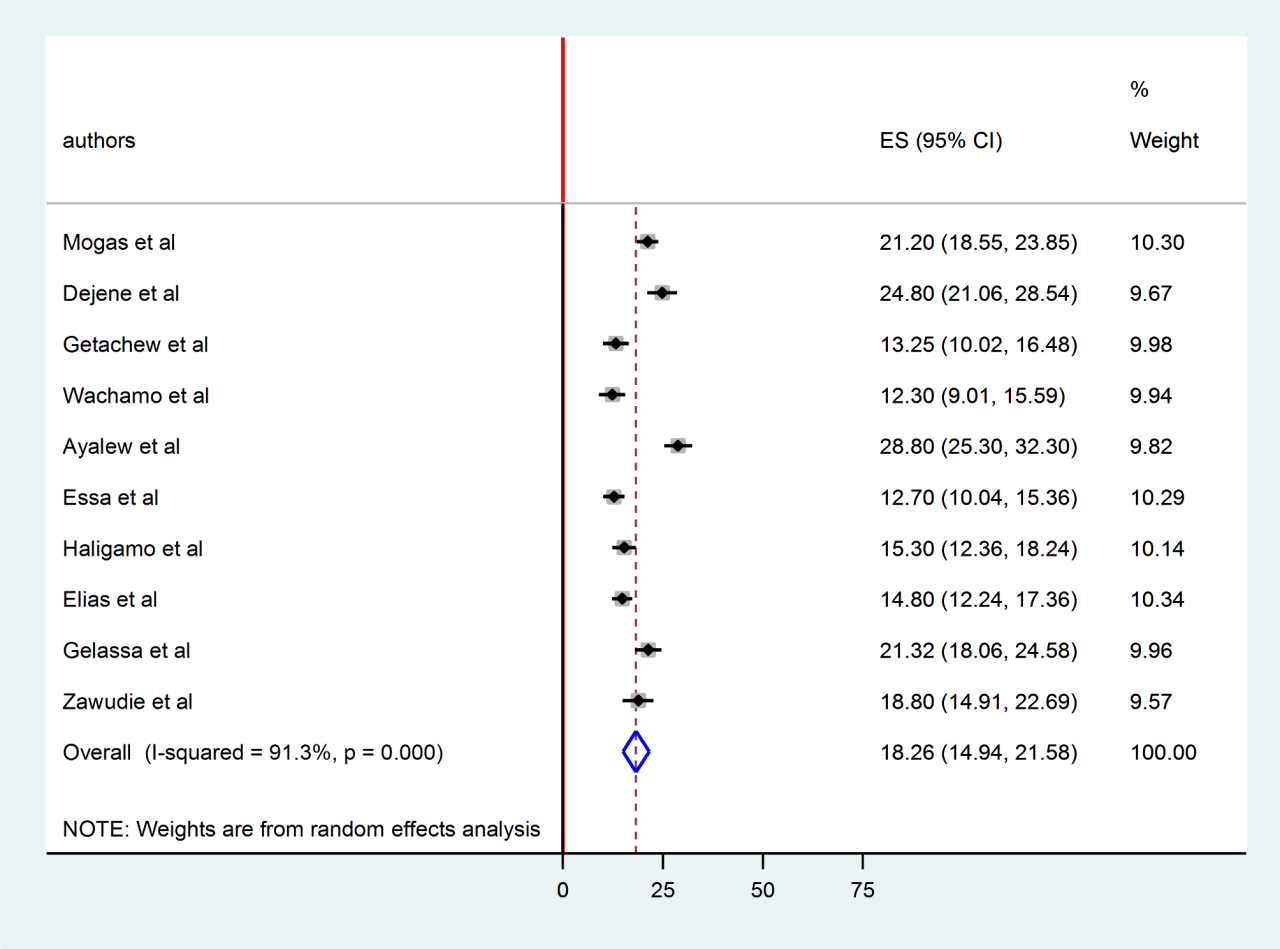
forest plot displaying pooled prevalence undiagnosed hypertension among adults in Ethiopia, 2023

The pooled prevalence of undiagnosed hypertension among adults in Ethiopia was 18.26% (95% CI 14.94 21.58). As shown in the forest plot below, statistically significant heterogeneity was observed (I^2^ = 91.3%; p < 0.0001). Therefore, we estimated the pooled prevalence of undiagnosed hypertension using random effects models (Fig. 2). In terms of individual prevalence, Sidama region had the lowest (12.3%) [27] and SNNPR had the highest (28.8%) prevalence of undiagnosed hypertension[30].

### Subgroup analysis

**Fig. 3:**
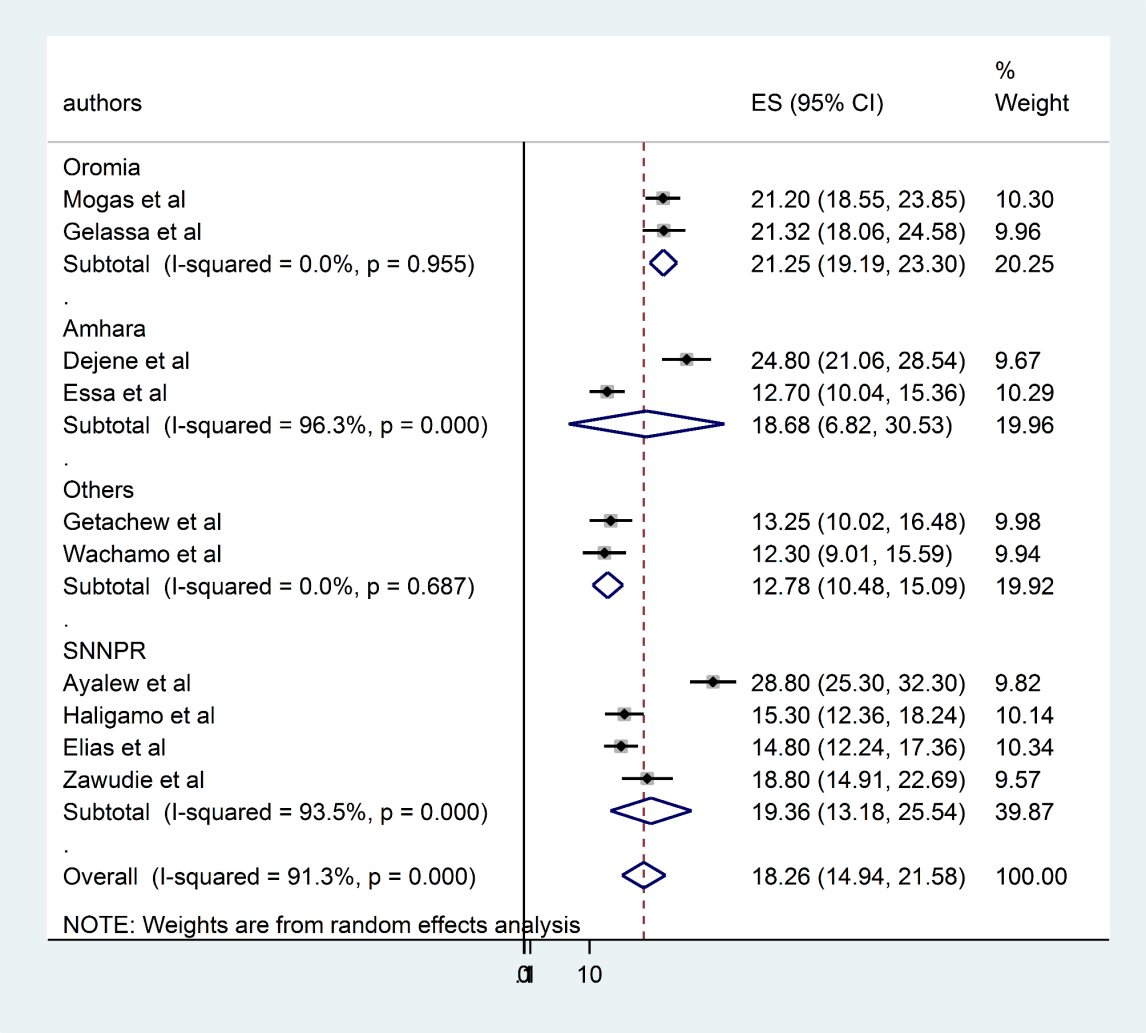
forest plot displaying subgroup analysis on the pooled prevalence of undiagnosed hypertension by region among adults in Ethiopia, 2022

**Fig. 4:**
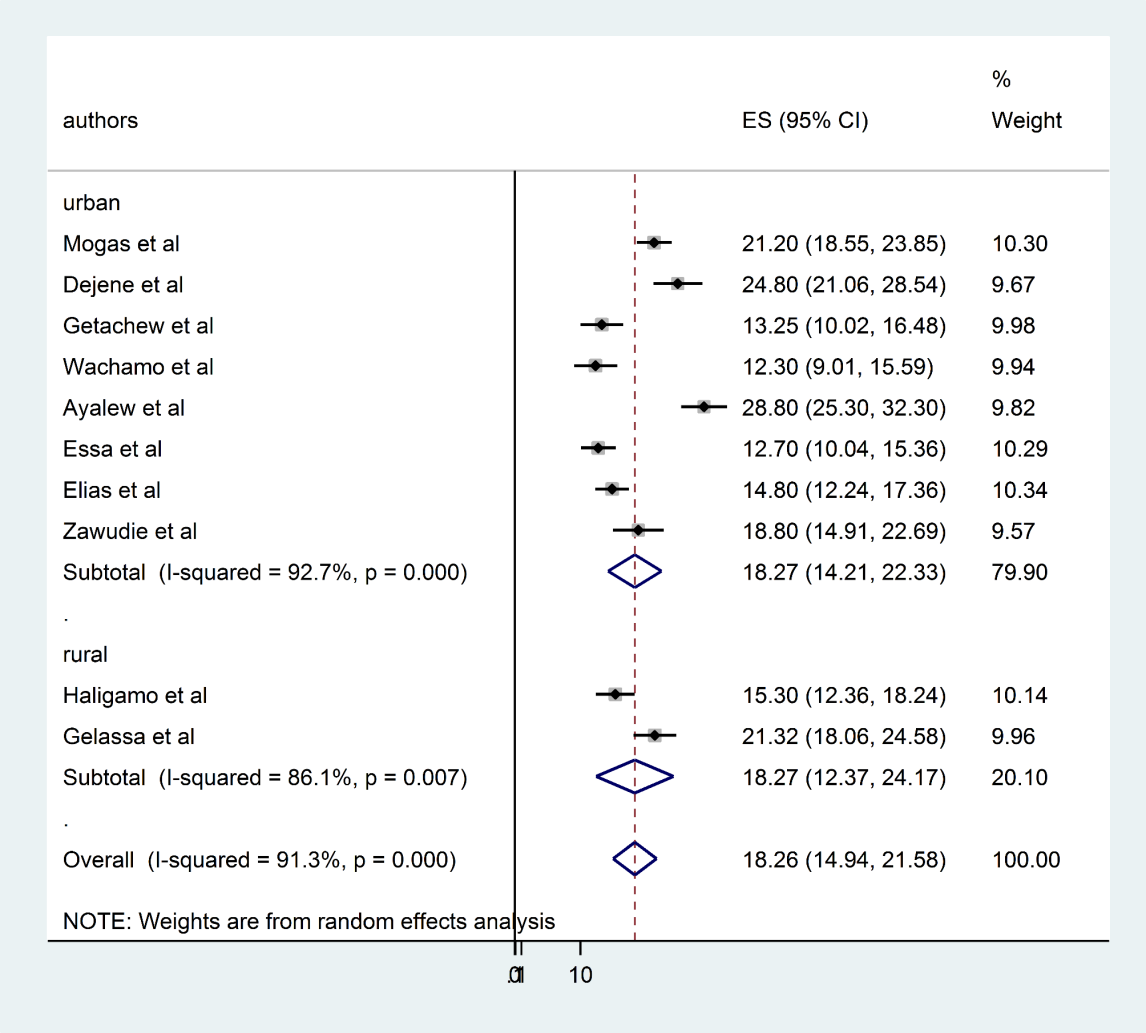
forest plot displaying subgroup analysis by study setting for the pooled undiagnosed hypertension among adults in Ethiopia, 2023

To assess the possible sources of heterogeneity, a subgroup analysis was performed based on the study area (regions) and study setting (urban/rural). Based on the subgroup analysis by region of Ethiopia the highest prevalence of undiagnosed hypertension was reported in Oromia region 21.51% (95% CI = 69.82, 79.30) and the lowest was in Amhara 15.92% (95% CI = 32.82, 62.41) (Fig. 3). According to subgroup analysis by study setting the pooled prevalence of undiagnosed hypertension among adults was similar 18.27% (95% CI = 14.21, 22.33) and 18.27% (95% CI = 12.94, 21.58) in urban and rural studies (Fig. 4).

### Heterogeneity and publication bias

**Fig. 5:**
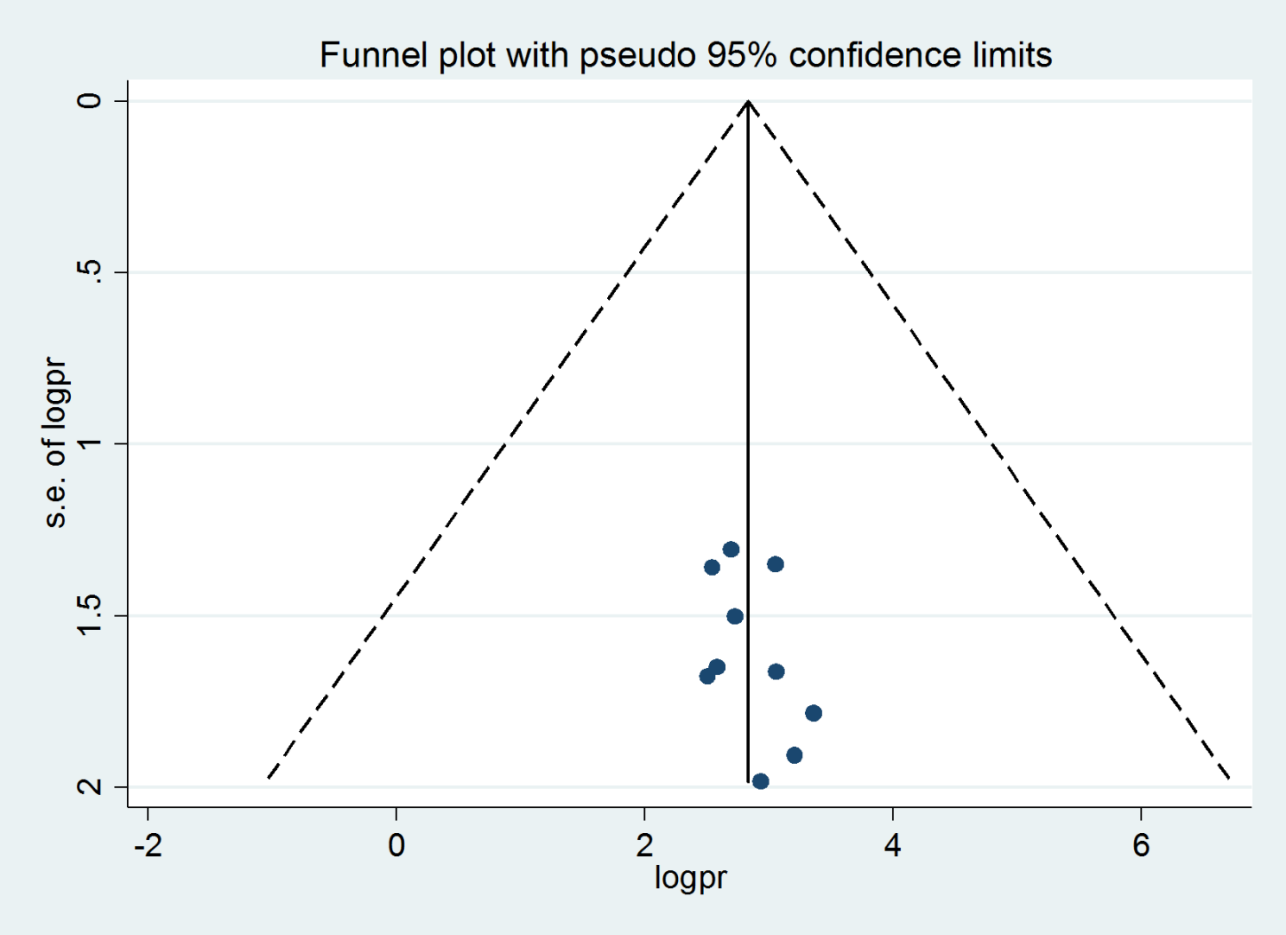
funnel plot displaying publication bias of studies reporting the pooled undiagnosed hypertension among adults in Ethiopia, 2023

The funnel plot was visually inspected to assess potential publication bias, which was statistically supported by the Begg’s and Egger’s tests. The symmetrical distribution of the included publications in a large inverted funnel indicated the absence of a publication bias(Fig. 5). The Begg and Egger tests revealed no publication bias among the studies included to estimate the pooled prevalence of undiagnosed hypertension among adults in Ethiopia, with p - values of (p = 0.65) and (p = 0.75), respectively.

### Sensitivity analysis

**Fig. 6:**
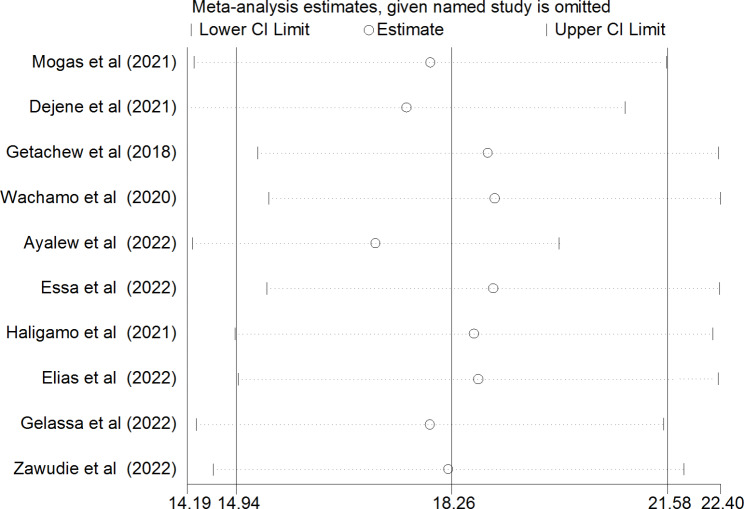
Sensitivity analysis on the studies included in systematic review and meta-analysis on prevalence of undiagnosed hypertension by region among adults in Ethiopia, 2023

Sensitivity analysis was used to assess the effect of a single study on the pooled undiagnosed hypertension among adults in Ethiopia by excluding each study one at a time. The findings revealed that no single study made a statistically significant difference in the pooled undiagnosed hypertension among adults in Ethiopia (Fig. 6).

### Determinants of undiagnosed hypertension among adults

To identify factors associated with undiagnosed hypertension, variables such as age, sex, family history of hypertension, body mass index, and presence of DM comorbidity, dietary practice and triglyceride level were extracted from the included studies. Finally, four variables were identified as independent predictors of undiagnosed hypertension among adults: age, family history of hypertension, body mass index and presence of DM comorbidity.

**Fig. 7:**
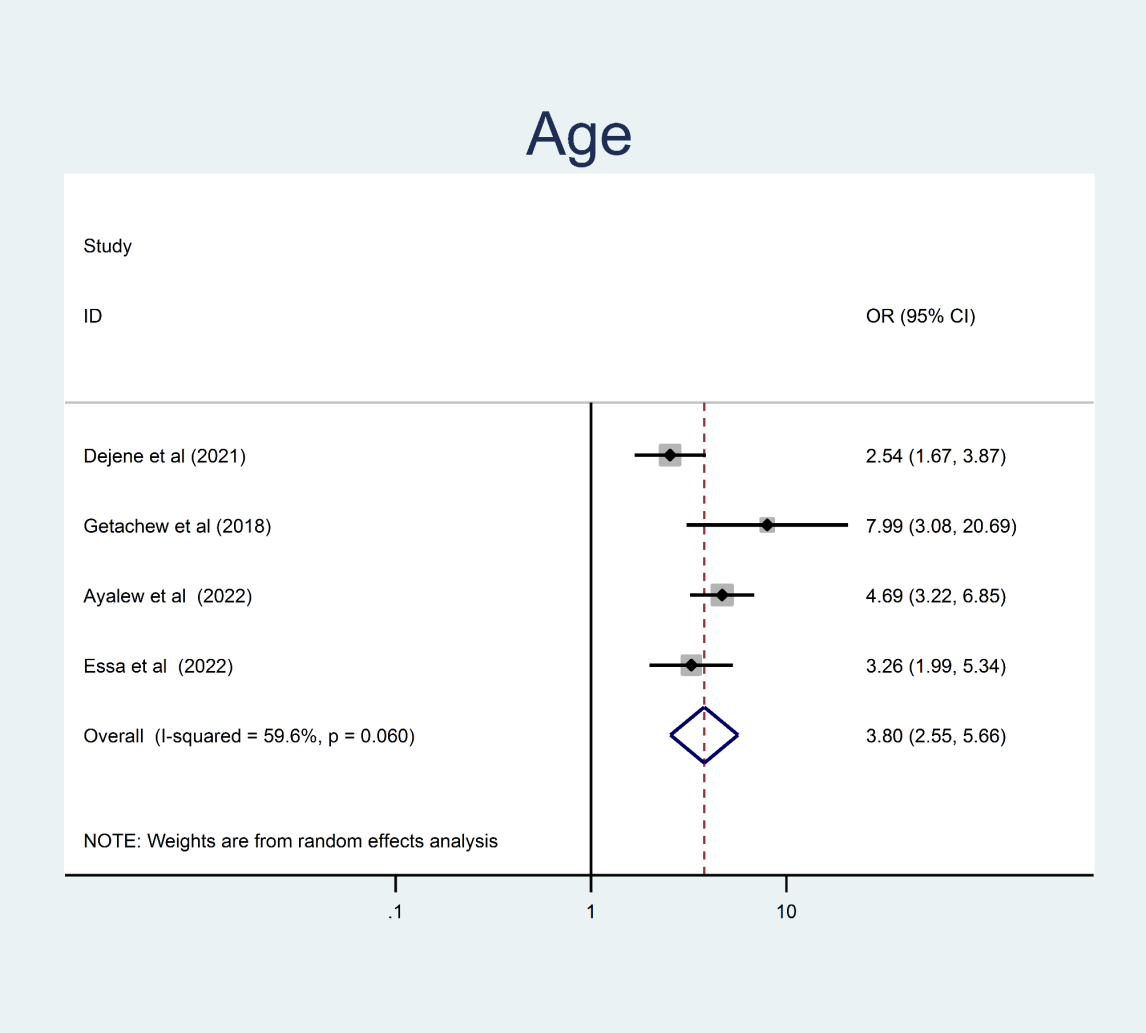
The pooled odd ratio of the association between age and undiagnosed hypertension among adults in Ethiopia, 2023

**Fig. 8:**
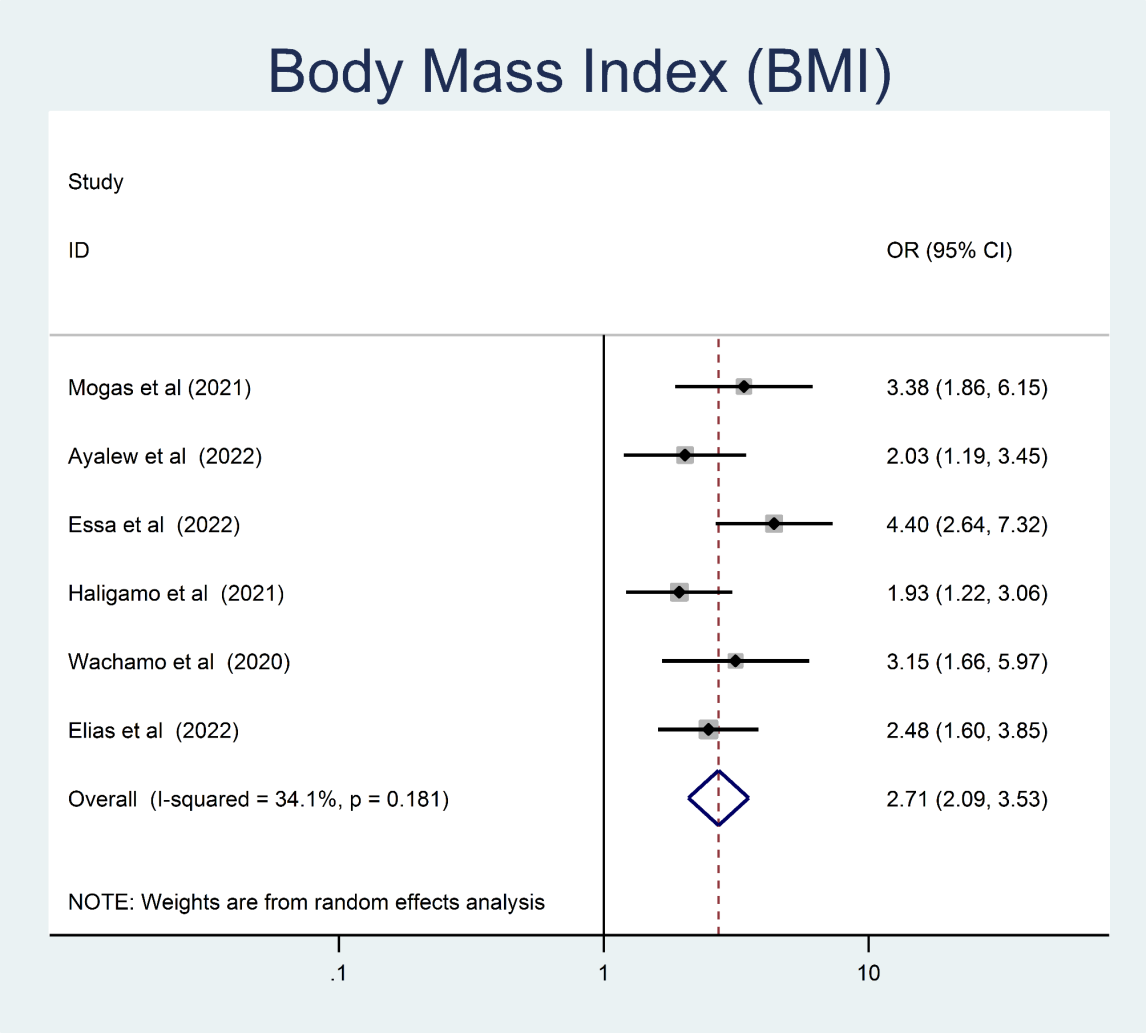
The pooled odd ratio of the association between body mass index and undiagnosed hypertension among adults in Ethiopia, 2023

In this study, being older age were 3.8 times more likely to develop undiagnosed hypertension than younger study participants (OR = 3.8, 95% CI = 2.56, 5.66) (Fig. 7). The odds of undiagnosed hypertension were 2.71 times higher among obese/overweight adult as compared to those who had normal body mass index (OR = 2.71, 95% CI = 2.1, 3.53) (Fig. 8).

**Fig. 9:**
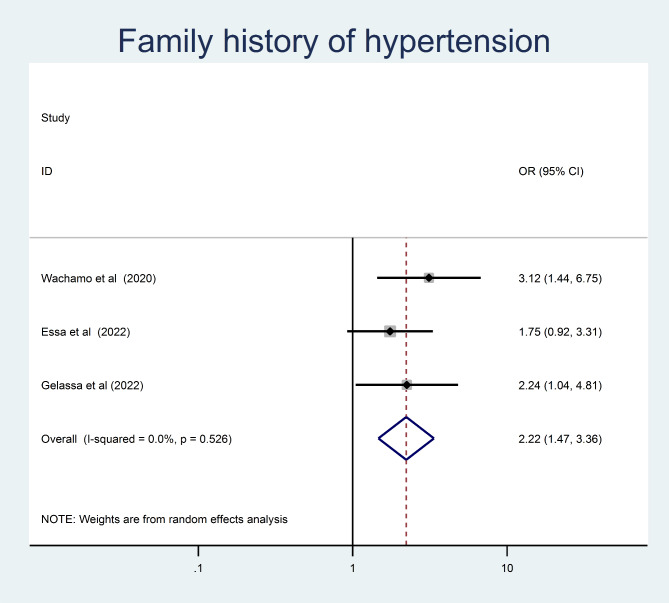
The pooled odd ratio of the association between family history of hypertension and undiagnosed hypertension among adults in Ethiopia, 2023

**Fig. 10:**
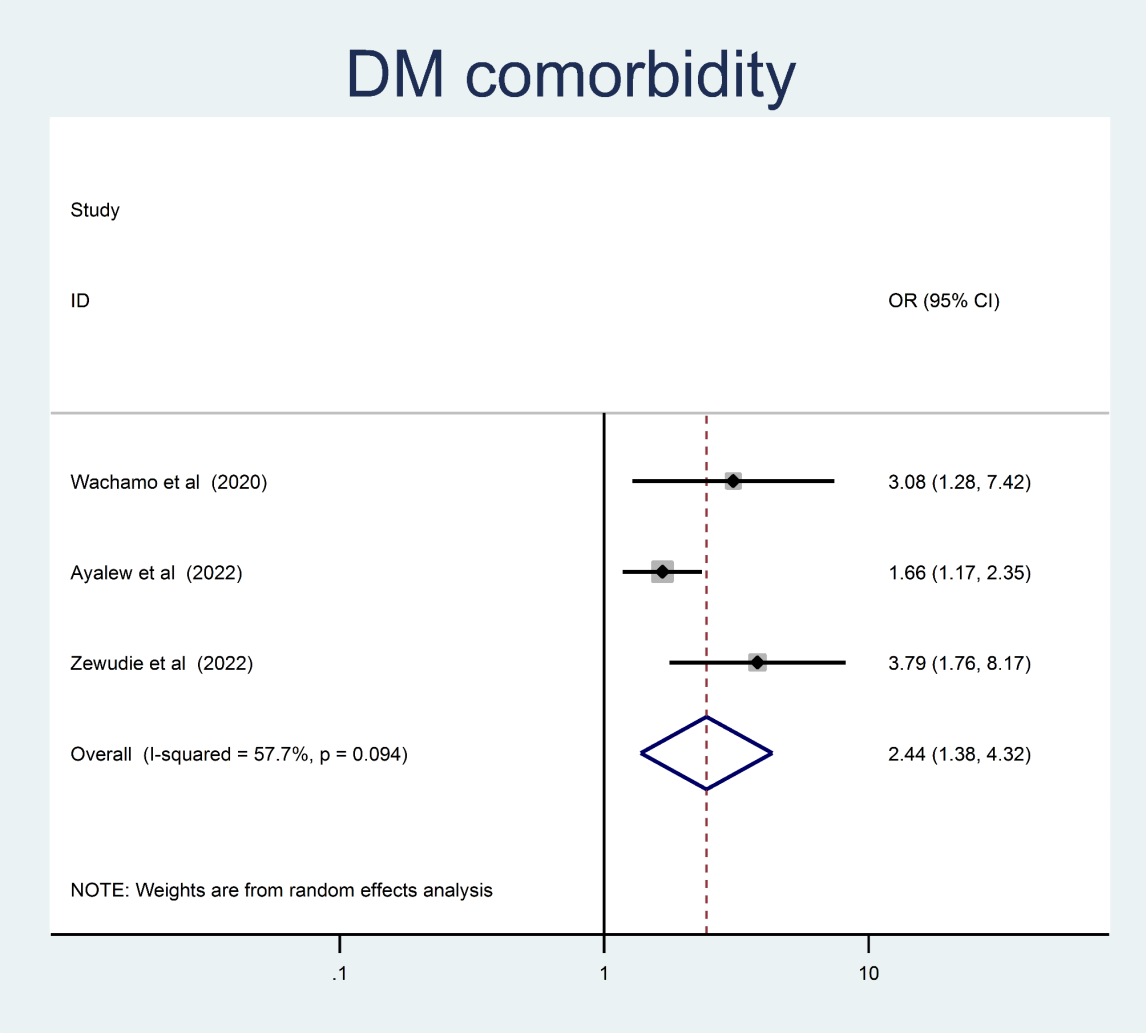
The pooled odd ratio of the association between presence of DM comorbidity and undiagnosed hypertension among adults in Ethiopia, 2023

Adults who had a family history of hypertension were 2.22 times more likely to develop than those who did not (OR = 2.22, 95% CI = 1.47, 3.36) (Fig. 9). Moreover, adults with a known DM comorbidity had a 2.44 times higher chance of being hypertensive than those who hadn’t (OR = 2.44, 95% CI = 1.38, 4.32) (Fig. 10).

## Discussion

There is evidence that the prevalence of hypertension is rising rapidly in many developing countries, and that undiagnosed hypertension is more likely to be linked to severe hypertension which leads to stroke, cardiovascular disease, and ischemic heart disease[7]. Therefore, this systematic review and meta-analysis aimed to provide an updated pooled prevalence of undiagnosed hypertension among adults in Ethiopia.

The current systematic review and meta-analysis revealed that the pooled prevalence of undiagnosed hypertension in Ethiopia was 18.26% (95% CI 14.94, 21.58). This finding is comparable with those studies conducted on rural Ruanda18.7% [34], Chinese and Korean American immigrants (14.5%) [35], and United Kingdom patients (17%) [36]. This finding implies that strengthening the identification rate for undiagnosed hypertension is required. However, the predicted epidemic in developing nations and global rise in the trend of hypertension will be controlled.

However, the pooled prevalence of undiagnosed hypertension in this meta-analysis was lower than that systematic review and meta-analysis conducted in SSA 30% [37]. Moreover it was also lower than the primary studies conducted in South Africa 50% [38], Nigeria 23.3% [39],, Central Africa Republic 69.9% [40], Sudan national survey 26.2% [41], Nigerian university community 27.8% [42] and Bangladesh 59.9% [19]. The lower prevalence observed in the current study might be due to the differences in the sociodemographic characteristics of the study participants between study participants in terms of their socio-demographic characteristics. For example, the systematic review and meta-analysis in SSA were performed on population with greater venerability.

On the other hand the pooled prevalence of undiagnosed hypertension in this meta-analysis was higher than primary study conducted in United States (4.5%) [43]. The higher prevalence of undiagnosed hypertension in our study is likely due to a combination of factors related to lack routine health screening, limited access to health care, low awareness, and some cultural beliefs that may discourage peoples to seek medical care in developing countries. Therefore, addressing these factors will be important in improving early detection and management of hypertension.

According to a subgroup analysis by study setting the pooled prevalence of undiagnosed hypertension among adults was similar (18.27%). The findings supported by studies conducted in the United States 25.5% in rural areas and 25.7% in urban areas [44] and in India 83.2% in rural and 85.3% in urban areas [45]. This could be due to the number of studies included in the analysis in which only two studies was represented rural communities. In addition the possible reasons for the similarity in the prevalence of undiagnosed hypertension between rural and urban areas could be due to lack of awareness and education about hypertension in both settings, and there may be limited access to medical care, particularly in rural areas, which can prevent individuals from receiving a diagnosis.

In the subgroup analysis the prevalence of undiagnosed hypertension varied significantly across the subnational region of Ethiopia. Based on this subgroup analysis the highest prevalence undiagnosed hypertension was from Oromia 21.51% followed by SNNPR and the lowest was from Amhara 15.92% The possible explanation could be due to the difference in study area and as well as cultural variations on feeding practice, lifestyle and the number of studies include in the analysis in which Oromia regional state of Ethiopia were represented by only two studies.

This systematic review and meta-analysis identified risk factors for undiagnosed hypertension among adults in Ethiopia. The age of participants was significantly associated with the development of hypertension. The pooled odds of developing undiagnosed hypertension were approximately four times higher among the older adults than among the younger study participants. This finding is supported by studies of Chinese and Korean American Immigrants [35], Bangladesh [19], the Central Africa Republic [40], Nigeria [39] and Sudan national survey .[41] This is explained by the fact that as people agedthey are more likely to develop chronic illnesses such as hypertension. In older patients, arterial walls may stiffen, blood vessel viscoelasticity may decrease, and blood flow resistance may increase [41].

A family history of hypertension was also associated with a higher prevalence of undiagnosed hypertension. This was supported by a studies conducted in Jordan [46] and Sri Lanka [47]. Hereditary factors that increase the risk of high blood pressure may contribute to the higher risk of undiagnosed hypertension in those with a family history of the condition.

The study showed that adult patients with DM comorbidity were significantly more likely to have undiagnosed hypertension. The current finding is consistent with a studies conducted in Sudan rural community [48] and United states of America [49]. An alternative explanation is that blood vessels experience extensive damage as blood glucose level increase. As a result, the body’s fluid volume increases, blood vessels lose some of their elasticity, and insulin resistance may increase the chance of developing high blood pressure. Hyperglycemia can increase blood volume, overload the kidneys, cause sodium and water retention, and ultimately raise blood pressure. A disturbance in glucose metabolism may hasten the hardening of the renal artery and systemic arterioles and increase peripheral resistance [50].

Our study found that obese individuals were 2.7 times higher likely to develop undiagnosed hypertension than those with a normal BMI. This was supported by primary studies in Central Africa Republic [40], Nigeria [39], Lebanon [51], and Sudan national survey [41]. Several mechanisms underlying the relationship between obesity and hypertension [52]. One mechanism could be that leptin, an adipokine found in higher levels in obese individuals, acts in the hypothalamus to increase blood pressure through activation of the sympathetic nervous system [53]. Therefore, to reduce and track the occurrence of undiagnosed hypertension among Ethiopians, educational campaigns to promote awareness of maintaining a healthy weight based on the BMI criteria are crucial.

### Limitation of the study

Although this is the first study to examine the pooled prevalence of undiagnosed hypertension and its associated factors in Ethiopia, the limitations stated below should be considered when interpreting the results. All the included studies were cross-sectional, which, owing to the nature of the study design, makes it challenging to demonstrate a cause-and-effect relationship. In addition, the investigations were restricted to only five regions, which would reduce the findings’ representativeness. Last but not least, we were forced to compare our findings with those of primary studies due to a lack of sufficient regional and worldwide systematic reviews and meta-analyses.

## Conclusion

The pooled prevalence of undetected hypertension was noticeably high in this review. The existence of DM comorbidity, BMI > 25 kg/m2, older age, and family history of hypertension were identified to be risk factors for undetected hypertension. In light of this, we recommend developing a screening program that focus on the elderly, those with hypertension family histories, adult obese and those with diabetes comorbidity. This will help with early diagnosis and prevent premature death from complications brought on by undiagnosed hypertension.

## Acronyms

BMI ; body mass index, CI; Confidence Interval, DM; diabetes mellitus, JBI; Joanna Briggs Institute, NCDs; Non-communicable Diseases, OR; Odds Ratio, PRISMA, Preferred Reporting Items for Systematic Reviews and Meta-Analyses, SSA; Sub-Sahara Africa, SNNPR; Southern Nation Nationalities and People Region, STEPS; STEP-wise approach to NCD risk factor surveillance, WHO; word health organization,

## Electronic supplementary material

Below is the link to the electronic supplementary material.

Supplementary Material 1

## Data Availability

All relevant data used for the systematic review and meta-analysis are within the manuscript and its supporting information.
